# Multiple imputation of covariates by fully conditional specification: Accommodating the substantive model

**DOI:** 10.1177/0962280214521348

**Published:** 2015-08

**Authors:** Jonathan W Bartlett, Shaun R Seaman, Ian R White, James R Carpenter, for the Alzheimer's Disease Neuroimaging Initiative*

**Affiliations:** 1 Department of Medical Statistics, London School of Hygiene & Tropical Medicine, UK; 2 MRC Biostatistics Unit, Cambridge, UK; 3 MRC Clinical Trials Unit, London, UK

**Keywords:** multiple imputation, compatibility, non-linearities, interactions, rejection sampling, fully conditional specification

## Abstract

Missing covariate data commonly occur in epidemiological and clinical research, and are often dealt with using multiple imputation. Imputation of partially observed covariates is complicated if the substantive model is non-linear (e.g. Cox proportional hazards model), or contains non-linear (e.g. squared) or interaction terms, and standard software implementations of multiple imputation may impute covariates from models that are incompatible with such substantive models. We show how imputation by fully conditional specification, a popular approach for performing multiple imputation, can be modified so that covariates are imputed from models which are compatible with the substantive model. We investigate through simulation the performance of this proposal, and compare it with existing approaches. Simulation results suggest our proposal gives consistent estimates for a range of common substantive models, including models which contain non-linear covariate effects or interactions, provided data are missing at random and the assumed imputation models are correctly specified and mutually compatible. Stata software implementing the approach is freely available.

## 1 Introduction

Missing data are a pervasive problem in both experimental and observational medical research, causing a loss of information and potentially biasing inferences. In this article, we focus on settings in which interest lies in fitting a substantive model relating an outcome to a number of covariates, one or more of which have missing values. The method of multiple imputation (MI) has become an extremely popular approach for accommodating missing data in statistical analyses, both generally^1^ and in the specific context of partially observed covariates.^2^ MI involves ‘filling in’ each missing value with draws from an appropriate distribution, leading to a number *M* of completed datasets. The substantive model can then be fitted to each of the *M* completed datasets, and the results combined across the *M* datasets using Rubin’s rules,^3^ which account for the uncertainty due to the fact data have been imputed. MI is most often applied under the missing at random (MAR) assumption, which stipulates that the probability that data are missing are independent of the missing values, conditional on the observed data, although MI can also be used when data are missing not at random.^3^

Parametric MI as originally proposed is based on a joint imputation model for the partially observed variables (conditional on any fully observed variables), which we refer to as ‘joint model MI’. A popular alternative to joint model MI is the fully conditional specification (FCS) approach.^4,5^ FCS MI involves specifying a series of univariate models for the conditional distribution of each partially observed variable given the other variables. This permits a great deal of flexibility, since an appropriate regression model can be selected for each variable (e.g. linear regression for continuous variables, logistic regression for binary variables). Consequently, FCS MI is particularly appealing in settings in which a number of variables have missing data, some of which are continuous and some of which are discrete.

One of the strengths of MI is that it divides the process of dealing with missingness (the imputation stage) from the analysis of the completed data (the analysis stage). As has been previously discussed in detail, this division presents both opportunities and threats.^6–9^. For example, we may be able to include so-called auxiliary variables in the imputation model which are not used in the analysis stage. This offers the potential for increased efficiency and may also improve the plausibility of the MAR assumption holding, by conditioning on auxiliary variables which are predictive of missingness. Sometimes the imputer and analyst will be different people. If the imputer has additional knowledge which enables him to impose (correct) additional assumptions in the imputation model, the analyst will gain efficiency.

The division may however sometimes lead to problems. In the context of imputing partially observed covariates, imputations might be generated from a model which is *incompatible* with the substantive model, which may lead to (asymptotically) biased estimates of parameters in the latter. Two conditional models are said to be incompatible if there exists no joint model for which the conditionals (for the relevant variables) equal these conditional models. This is a particular issue when the substantive model contains non-linear covariate effects or interactions, with which default imputation model choices may be incompatible. For example, suppose the substantive model is the linear regression of *Y* on a continuous covariate *X* and *X*^2^, and we wish to impute missing values in *X*. The default choice for the imputation model for X|Y in software for MI is a normal linear model, with conditional mean equal to a linear function of *Y*, which is incompatible with the quadratic substantive model. Following imputation of *X*, *X*^2^ is then passively imputed by squaring the imputed *X* values. In this case, estimates of the parameters of the substantive model from multiply imputed datasets will be biased (unless the quadratic coefficient is in truth zero), because within the subset of data where *X* has been imputed the association between *X* and *Y* is linear as a consequence of assuming linearity in the imputation model.^10^

Incompatibility between the imputation and substantive models can be avoided by specifying a joint model for outcome and covariates for which the conditional distribution of outcome given covariates matches the substantive model and then using the imputation model implied by this joint model. However, specification of a joint model is challenging when there are a number of partially observed covariates, particularly when some are continuous and some are discrete. In this setting, the FCS method is an attractive option. However, the default univariate imputation models used in FCS may be incompatible with the substantive model. In this article, we therefore propose a modification of the popular FCS approach to MI which ensures that each of the univariate imputation models is compatible with the assumed substantive model.

We begin in Section 2 by introducing a motivating example from the Alzheimer’s Disease Neuroimaging Initiative (ADNI). In Section 3, we formally define compatibility between an imputation model for partially observed covariates and a substantive model, explain when incompatibility implies imputation model mis-specification and give examples of when this occurs. We then outline how imputation models can be specified which are compatible with a given substantive model within the joint modelling approach to MI, which motivates our modification of the FCS MI approach. In Section 4, we briefly review the standard FCS framework for MI in the setting of partially observed covariates. In Section 5, we describe our modification of the FCS MI approach which ensures that each univariate imputation model is compatible with the substantive model. In Section 6 we give details for how this can be done when the model of interest is (i) normal linear regression, (ii) a model for a discrete outcome (e.g. logistic and Poisson regression) or (iii) a proportional hazards model. We report the results of a simulation study to investigate the performance of our proposed approach in Section 7. In Section 8, we apply our proposed approach to the motivating example. In Section 9, we discuss how our proposed approach can be used when, as is often the case, interest lies in fitting a number of different substantive models to the data. We conclude in Section 10 with a discussion.

## 2 Motivating example

The ADNI was launched in 2003 by the National Institute on Aging (NIA), the National Institute of Biomedical Imaging and Bioengineering (NIBIB), the Food and Drug Administration (FDA), private pharmaceutical companies and nonprofit organisations, as a 5-year public–private partnership. The aims of ADNI included assessing the ability of imaging and other biomarkers to measure the progression of mild cognitive impairment and early Alzheimer’s disease (AD). The study aimed to recruit approximately 200 cognitively normal older individuals (controls), 400 with mild cognitive impairment (MCI) and 200 with early AD. Participants underwent clinical and cognitive assessment and MRI brain scans at baseline and at specified intervals (every 6 or 12 months, depending on subject group) up to 3 years. Further details regarding ADNI are given in the acknowledgements.

Recently Jack et al*.*^11^ used data from ADNI to investigate baseline predictors of time to conversion to AD in those subjects with MCI at baseline. In particular, using Cox proportional hazards models they found evidence of a non-linear association between amyloid β 1-42 peptides (A$\beta_{1-42}$) measured from cerebrospinal fluid (CSF) at baseline and log hazard of conversion. They found evidence that lower baseline hippocampal volume was predictive of increased hazard, after adjusting for total intracranial volume (a measure of head size). Jack et al*.*^11^ also found evidence that the presence of the APOE4 gene, previously shown to be associated with development of AD in a number of studies, was associated with increased hazard for conversion to AD.

Participants were invited to have CSF measured at baseline by lumbar puncture, but were not required to do so to participate in the study. Consequently, only around 50% of subjects had CSF A$\beta_{1-42}$ measured. The analysis of Jack et al*.*^11^ was restricted to the subset of *n* = 218 MCI subjects for whom CSF A$\beta_{1-42}$ was measured. In a logistic regression analysis we found no evidence that availability of CSF was related to first of time to conversion to AD or censoring, or to the event indicator (joint test *p* = 0.48). A second logistic regression model found evidence (*p* = 0.02) that having a family history of AD was associated with an increased probability of having the CSF variables recorded, which seems quite plausible since those with a family history of AD may be especially concerned about their risk of developing the disease. These results suggest a complete case analysis might reasonably be assumed to be unbiased. However, it is inefficient, since it only uses data on 50% of MCI subjects.

MCI is a heterogeneous classification, with only a certain proportion of subjects eventually going on to develop AD. For each subject their family history was collected at baseline, in particular in relation to whether their mother or father suffered from dementia or AD. Given that there is a genetic component to the disease, we were interested to investigate whether the presence of family history of AD was associated with increased hazard of conversion to AD, by including covariates indicating whether the subject’s mother and father had had AD (Table 1). Unfortunately, although family history of dementia was well recorded, family history of AD specifically suffered from missingness (see Table 1).

**Table 1.** Baseline characteristics of *n* = 382 ADNI subjects with MCI at baseline.

Variable	Mean (SD) or no. (% of observed)	No. of missing values (%)
A$\beta_{1-42}$ (ng/mL)	16.4 (5.5)	190 (49.7%)
log(tau) (log pg/mL)	4.50 (0.49)	193 (50.5%)
log(p-tau) (log pg/mL)	3.44 (0.50)	189 (49.5%)
Mother had AD	77 (25.3%)	77 (20.2%)
Father had AD	26 (9.0%)	93 (24.3%)
Intracranial volume (cm^3^)	1474 (150)	43 (11.3%)
Hippocampal volume (cm^3^)	6.47 (1.04)	43 (11.3%)
APOE4 positive	207 (54.2%)	0 (0%)

ADNI: Alzheimer’s Disease Neuroimaging Initiative; MCI: mild cognitive impairment; AD: Alzheimer’s disease.

We aimed to estimate the parameters of a Cox proportional hazards model for hazard of conversion to AD using the available data from the *n* = 382 ADNI subjects who had MCI at baseline and who had at least one follow-up visit. Of these subjects, 167 were observed to convert to AD during follow-up. Our substantive model contained as covariates the variables listed in Table 1, plus the square of A$\beta_{1-42}$ to allow for the non-linear association previously identified by Jack et al*.*^11^. In addition to CSF A$\beta_{1-42}$, we included CSF tau and p-tau as covariates, which are also thought to reflect pathology, and thus might be associated with hazard of conversion to AD. Tau and p-tau were log transformed to reduce skewness in their distribution. The FCS approach to MI is attractive here, since seven covariates are partially observed, with some continuous and some binary. However, we should be careful to ensure that the imputation models we use are compatible with the substantive model, which includes a quadratic effect of one of the partially observed covariates. If we impute from a model which does not allow for a potential non-linear association between (log) hazard and CSF A$\beta_{1-42}$, we would expect to obtain inconsistent parameter estimates, particularly of the coefficients relating to CSF A$\beta_{1-42}$.

## 3 MI of partially observed covariates

### 3.1 Setup

We consider the setting in which interest lies in fitting a model to a fully observed outcome *Y* (although see Section 10) with *p* partially observed covariates $X=(X_{1},\ldots ,X_{p})$ and *q* fully observed covariates $Z=(Z_{1},\ldots ,Z_{q})$. Let *X^obs^* and *X^mis^* denote the observed and missing components of *X* for a given subject, and let *R* be the vector of observation indicators whose elements are zero or one depending on whether the corresponding element of *X* is missing or observed. We assume throughout that the data are MAR.^12^ Here, MAR means that P(R|Y,X,Z)=P(R|Y,Xobs,Z). We assume that $(Y_{i},X_{i},Z_{i},R_{i}),i=1,\ldots ,n$ are independent and identically distributed. Lastly, we let f(Y|X,Z,ψ) denote the ‘substantive model’, which is indexed by parameter ψ (ψ∈Ψ). We assume throughout that this substantive model is correctly specified. That is, there exists ψ∈Ψ such that $f_{0}(Y|X,Z)=f(Y|X,Z,\psi )$, where $f_{0}(Y|X,Z)$ denotes the true conditional distribution of *Y* given *X* and *Z*.

### 3.2 MI of partially observed covariates

In Bayesian parametric MI, to multiply impute missing values in *X* we specify a parametric model f(X|Z,Y,ω),ω∈Ω for the conditional distribution f(X|Y,Z). To create the *m*th imputed dataset we first draw ω(m) from its posterior distribution given the observed data {(Y,Xobs,Z);i=1,…,n} and a (usually noninformative) prior *f*(ω). For each subject the missing values (if any) *X^mis^* are imputed by taking a draw from the density f(Xmis|Xobs,Y,Z,ω(m)) implied by f(X|Y,Z,ω(m)).

Having created *M* imputed datasets, the substantive model parameter ψ is then estimated separately using each imputed dataset, resulting in estimates $\overset{\wedge }{\psi }1,\ldots ,\overset{\wedge }{\psi }M$ and corresponding variances $\overset{\wedge }{\text{Var}}(\overset{\wedge }{\psi }1),\ldots ,\overset{\wedge }{\text{Var}}(\overset{\wedge }{\psi }M)$. In this article, we assume that the substantive model is fitted using maximum likelihood. Rubin’s rules are then invoked to provide a final inference for ψ, with the estimator of ψ given by

$${\overset{\wedge }{\psi }}_{\mathrm{MI}}=\frac{\sum_{m=1}^{M}\overset{\wedge }{\psi }m}{M}$$

and an estimate of the variance of ${\overset{\wedge }{\psi }}_{\mathrm{MI}}$ given by

$$\overset{\wedge }{\text{Var}}({\overset{\wedge }{\psi }}_{\mathrm{MI}})=[\frac{1}{M}\sum_{m=1}^{M}\overset{\wedge }{\text{Var}}(\overset{\wedge }{\psi }m)]+[(1+1/M)\frac{1}{M-1}\sum_{m=1}^{M}(\overset{\wedge }{\psi }m-{\overset{\wedge }{\psi }}_{\mathrm{MI}})2]$$

Suppose that the posited imputation model is correctly specified, so that there exists a value of ω∈Ω such that $f_{0}(X|Y,Z)=f(X|Z,Y,\omega )$. Then ${\overset{\wedge }{\psi }}_{\mathrm{MI}}$ is a consistent estimator of ψ, and as the number of imputations M→∞, confidence intervals (CIs) based on $\overset{\wedge }{\text{Var}}({\overset{\wedge }{\psi }}_{\mathrm{MI}})$ achieve coverage at or above their nominal level.^6^

### 3.3 Compatibility and imputation model mis-specification

When an imputation model f(X|Z,Y,ω) is directly specified, it may be mis-specified if it is not *compatible* with the substantive model (assuming this is correctly specified). For example, if the correctly specified substantive model includes an interaction between a partially observed covariate and a fully observed covariate in their effect on the outcome *Y*, imputation models which do not allow for this interaction will generally (unless the interaction term is in truth zero) be mis-specified. Such considerations led to recommendations that imputation models be used which do not impose restrictions which will conflict with subsequent analyses of the imputed datasets.^6,7^

Extending a definition given by Liu et al.,^13^ we now define the notion of compatibility between a set of conditional models. Let $A=(A_{1},\ldots ,A_{p})$ be a vector of random variables, and let $A_{-j}=(A_{1},\ldots ,A_{j-1},A_{j+1},\ldots ,A_{p})$. Let *B* denote a further, possibly empty, vector of random variables. Then a set of conditional models $\left\{f_{j}(A_{j}|A_{-j},B,\theta_{j});\theta_{j}\in \Theta_{j},j=1,\ldots ,p\right\}$ is said to be compatible if there exists a joint model g(A|B,θ),θ∈Θ and a collection of surjective maps $\left\{t_{j}:\Theta \to \Theta_{j};j=1,\ldots ,p\right\}$ such that for each *j*, $\theta_{j}\in \Theta_{j}$ and $\theta \in t_{j}^{-1}(\theta_{j})=\{\theta :t_{j}(\theta )=\theta_{j}\}$

$$f_{j}(A_{j}|A_{-j},B,\theta_{j})=g(A_{j}|A_{-j},B,\theta )$$

Otherwise the set of models $\left\{f_{j};j=1,\ldots ,p\right\}$ is said to be incompatible.

A weaker property which we shall also use is that of *semi-compatibility* for a set of models. A set of models is semi-compatible if they can be made compatible by setting one or more parameters to zero. More formally (again following Liu et al.^13^), a set of conditional models $\left\{h_{j}(A_{j}|A_{-j},B,\theta_{j},\kappa_{j});\theta_{j}\in \Theta_{j},\kappa_{j}\in K_{j},j=1,\ldots ,p\right\}$ is said to be semi-compatible if there exists a set of compatible conditional models $\left\{f_{j}(A_{j}|A_{-j},B,\theta_{j});\theta_{j}\in \Theta_{j},j=1,\ldots ,p\right\}$ such that

$$f_{j}(A_{j}|A_{-j},B,\theta_{j})=h_{j}(A_{j}|A_{-j},B,\theta_{j},\kappa_{j}=0)$$

for *j* = 1, … , *p*. A set of compatible conditional models is always semi-compatible. If the joint model g(A|B,θ) corresponding to the set of conditionals $\left\{f_{j};j=1,\ldots ,p\right\}$ is correctly specified (i.e. contains the true probability distribution), we say (again following Liu et al.^13^) that the set of models $\left\{h_{j};j=1,\ldots ,p\right\}$ is valid semi-compatible.

The imputation model f(X|Z,Y,ω) is correctly specified if and only if it is valid semi-compatible with the substantive model f(Y|X,Z,ψ). As an example, suppose the substantive model is $Y|X\;\sim N(\psi_{0}+\psi_{1}X+\psi_{2}X2,\sigma_{\psi }^{2})$ and the imputation model is $X|Y\;\sim N(\omega_{0}+\omega_{1}Y,\sigma_{\omega }^{2})$. These two models are incompatible (this can be established by the theorem for compatibility of two conditional densities of Arnold and Press^14^). They are semi-compatible, with the joint model for (*X, Y*) being the bivariate normal, by setting $\psi_{2}=0$. However, they are not valid semi-compatible, unless in truth $\psi_{2}=0$. The imputation model is therefore mis-specified in general, and the MI estimator ${\overset{\wedge }{\psi }}_{\mathrm{MI}}$ will be inconsistent, as demonstrated through simulation by von Hippel^15^ and Seaman et al.^10^

It is important to note that incompatibility between the imputation and substantive models does not necessarily imply mis-specification of the former. For example, suppose the substantive model is $Y|X\;\sim (\psi_{0}+\psi_{1}X,\sigma_{\psi }^{2})$ and the imputation model is $X|Y\;\sim N(\omega_{0}+\omega_{1}Y+\omega_{2}Y2,\sigma_{\omega }^{2})$, with each of the regression coefficients lying in (-∞,+∞). Then again the two models are incompatible, but are semi-compatible, with joint model the bivariate normal, by setting *ω*_2_ = 0. If the data in truth are bivariate normal, the two models are valid semi-compatible, and the imputation model is correctly specified. Here, incompatibility between imputation and substantive models does not imply mis-specification of the former because a more restrictive version of the imputation model (with *ω*_2_ = 0) is compatible with the substantive model.

Incompatibility may also arise when default imputation models are used for covariates in non-linear substantive models. For example, suppose *T* (rather than *Y*) is a time to event outcome which is not subject to censoring, and that the substantive model is the parametric exponential model, with hazard function $h(t)=h_{0}\exp(\psi X)$, with *X* a continuous partially observed covariate and *h*_0_ a time constant baseline hazard. In this case $H_{0}(T)=\int_{0}^{T}h_{0}\text{d}t=h_{0}T$. Then suppose, following the recent recommendations of White and Royston,^16^ we adopt a normal linear imputation model for $X|T\;\sim N(\omega_{0}+\omega_{1}T,\sigma_{\omega }^{2})$ with $T\propto H_{0}(T)$ as covariate. The two models are incompatible, and are only semi-compatible by setting *ω*_1_ = 0 and ψ=0, such that *T* and *X* are independent. The imputation model can thus only be valid semi-compatible (and thus correctly specified) with the substantive model if *T* and *X* are independent. The MI estimator ${\overset{\wedge }{\psi }}_{\mathrm{MI}}$ will thus generally be inconsistent (although simulations by White and Royston^16^ show the bias is often small).

In conclusion, except in cases where the imputation and substantive models can be made compatible by restricting the parameter space Ω of the imputation model, incompatibility between the two implies the imputation model is mis-specified (assuming correct specification of the substantive model). Consequently, when choosing the covariate imputation model f(X|Z,Y,ω) we should (at least) ensure that it is either compatible with the substantive model, or a restriction of it is compatible with the substantive model.

### 3.4 Joint model imputation

The natural route to ensuring compatibility between the imputation and substantive models is to explicitly specify a joint model g(Y,X|Z,θ) which has the substantive model f(Y|X,Z,ψ) as its corresponding conditional, and to derive the implied imputation model. Given the (correctly) specified substantive model f(Y|X,Z,ψ), such joint models are specified by defining a model f(X|Z,φ). The imputation model is then given by

(1) $$f(X|Z,Y,\psi ,\varphi )=\frac{f(Y,X|Z,\psi ,\varphi )}{f(Y|Z,\psi ,\varphi )}=\frac{f(Y|X,Z,\psi )f(X|Z,\varphi )}{f(Y|Z,\psi ,\varphi )}\propto f(Y|X,Z,\psi )f(X|Z,\varphi )$$

We emphasise that using a compatible imputation model does not guarantee it is correctly specified – this is only true if, in addition to the substantive model being correctly specified, f(X|Z,φ) is correctly specified.

In cases where *p* = 1 and *X* is univariate, specification of a model f(X|Z,φ) is relatively straightforward. When *X* is multivariate, and particularly when it contains a mixture of continuous and discrete variables, specification of a joint model f(X|Z,φ) becomes more challenging. In this setting, Ibrahim et al.^17^ proposed specification by factorising the joint distribution of X|Z as a product of univariate densities of the form

(2) $$f(X_{1}|Z)f(X_{2}|X_{1},Z)f(X_{3}|X_{1},X_{2},Z)\ldots .$$

This breaks the problem of joint specification into the easier task of specification of a series of univariate models. This means that appropriate univariate regression models can be specified depending on the type (i.e. continuous, discrete and ordered discrete) of each variable. However, as the dimension of *X* increases the number of possible orderings of its components increases rapidly, and it is not obvious which ordering should be chosen. As far as we are aware, this approach to MI has not been adopted by applied researchers. A new joint modelling approach for handling interactions and non-linearities has recently been proposed by Goldstein et al.^18^

## 4 FCS MI

In the more general setting of MI in multivariate data, the FCS approach to MI similarly splits the task of specification of a joint model into a series of univariate model specifications. In FCS MI, as we describe in further detail in Section 4.1, models are specified for each partially observed variable conditional on all other variables. In contrast to the approach proposed by Ibrahim et al.,^17^ there is no requirement to choose an ordering of the partially observed variables. FCS MI has now become an extremely popular approach to MI generally.^5^

Application of FCS for imputation of partially observed covariates involves specification of models of the form $f(X_{j}|X_{-j},Z,Y)$, where $X_{-j}$ denotes the components of *X* with *X_j_* removed. As we have described, for certain substantive models, such as those involving non-linear covariate effects or interactions, default choices of these imputation models within FCS will be incompatible. Further, they will not generally be valid semi-compatible with the substantive model, and will therefore be mis-specified (assuming correct specification of the substantive model). In the next sub-section, we review the standard FCS algorithm in detail, and then in Section 5 propose a modification of FCS MI which ensures that each of the covariate models $f(X_{j}|X_{-j},Z,Y)$ is compatible with the substantive model.

### 4.1 The FCS algorithm

For each partially observed covariate *X_j_*, we posit an imputation model $f(X_{j}|X_{-j},Z,Y,\theta_{j})$, with parameter θ*_j_*, where $X_{-j}=(X_{1},\ldots ,X_{j-1},X_{j+1},.\ldots X_{p})$. This is typically a generalised linear model chosen according to the type of *X_j_* (e.g. continuous, binary and count). Furthermore, a noninformative prior distribution *f*(θ*_j_*) for θ*_j_* is specified. Let $x_{j}^{\mathrm{obs}}$ and $x_{j}^{\mathrm{mis}}$ denote the vectors of observed and missing values in *X_j_* for the *n* subjects. Let *y* and *z* denote the vector and matrix of (fully observed) values of *Y* and *Z* across the *n* subjects.

The FCS algorithm begins by replacing the missing values in each *X_j_* by randomly selected observed values from the same variable. The algorithm then proceeds by repeatedly imputing the missing values in each variable, at each stage conditioning on the most recent imputations of the other variables. Let $x_{j}^{\mathrm{mis}(t)}$ denote the imputations of the missing values $x_{j}^{\mathrm{mis}}$ at iteration *t* and let $x_{j}^{\left(t\right)}=(x_{j}^{\mathrm{obs}},x_{j}^{\mathrm{mis}(t)})$ denote the vector of observed and imputed values at iteration *t*. Let $x_{-j}^{\left(t\right)}=(x_{1}^{\left(t\right)},\ldots ,x_{j-1}^{\left(t\right)},x_{j+1}^{\left(t-1\right)},\ldots ,x_{p}^{\left(t-1\right)})$. The *t*th iteration of the algorithm consists of drawing from the following distributions (up to constants of proportionality)

(3) $$\sim f(x_{1}^{\mathrm{mis}}|x_{-1}^{\left(t\right)},z,y,\theta_{1}^{\left(t\right)})\theta_{2}^{\left(t\right)}\sim f(\theta_{2})f(x_{2}^{\mathrm{obs}}|x_{-2}^{\left(t\right)},z,y,\theta_{2})x_{2}^{\mathrm{mis}(t)}\sim f(x_{2}^{\mathrm{mis}}|x_{-2}^{\left(t\right)},z,y,\theta_{2}^{\left(t\right)}):\theta_{p}^{\left(t\right)}\sim f(\theta_{p})f(x_{p}^{\mathrm{obs}}|x_{-p}^{\left(t\right)},z,y,\theta_{p})x_{p}^{\mathrm{mis}(t)}\sim f(x_{p}^{\mathrm{mis}}|x_{-p}^{\left(t\right)},z,y,\theta_{p}^{\left(t\right)})\}$$

Thus, for the partially observed covariate *X_j_* the algorithm first draws θ*_j_* from its posterior distribution given $x_{j}^{\mathrm{obs}},x_{-j}^{\left(t\right)},z,y$. This is equal (up to a constant of proportionality) to the product of the prior *f*(θ*_j_*) and the likelihood corresponding to fitting the imputation model for *X_j_* to subjects for whom *X_j_* is observed, using the observed and most recently imputed values of $X_{-j}$. Missing values in *X_j_* are then imputed from the imputation model using the parameter value drawn in the preceding step. After a sufficient number of iterations it is assumed that the algorithm has converged to a stationary distribution, and the final draws of the missing data form a single imputed dataset. The process is then repeated to create as many imputed datasets as desired. In software implementations of FCS MI, the variables are typically updated in the ordering for which the missingness pattern is closest to monotone. Finally, the substantive model is fitted to each imputed dataset, and the results combined using Rubin’s rules as described previously.

Note that when drawing θ*_j_*, the FCS algorithm differs from a standard Gibbs sampler, which would draw θ*_j_* from its posterior distribution proportional to $f(\theta_{j})f(x_{j}^{\mathrm{mis}(t-1)},x_{j}^{\mathrm{obs}}|x_{-j}^{\left(t\right)},z,y,\theta_{j})$, that is, the posterior corresponding to fitting the imputation model to all subjects, using the last iteration’s imputation of $X_{j}^{\mathrm{mis}},x_{j}^{\mathrm{mis}(t-1)}$.

### 4.2 Statistical properties

Despite the fact that FCS MI has been applied widely in a number of fields,^5^ until recently few results were available regarding its validity. This is due to the fact that one can specify imputation models (which in our setting are $f(X_{j}|X_{-j},Z,Y,\theta_{j})$) that are not mutually compatible.^19^ In this case, it is not clear to what distribution, if any, the algorithm will converge.

Hughes et al.^20^ have recently given conditions under which, at convergence, FCS draws imputations from a well-defined Bayesian joint model, which are as follows:

1. The conditional models $f(X_{j}|X_{-j},Z,Y,\theta_{j}),j=1,\ldots ,p$, are compatible, with corresponding joint model f(X|Z,Y,θ).
2. For each *j* = 1, … , *p*, this joint model can be factorised as $f(X|Z,Y,\theta )=f(X_{j}|X_{-j},Z,Y,\theta_{j})f(X_{-j}|Z,\theta_{-j})$, where $\left(\theta_{j},\theta_{-j}\right)$ is a reparameterisation of *θ*, and *θ_j_* and $\theta_{-j}$ are variationally independent.
3. There exists a prior *f*(*θ*) such that for each *j* = 1, … ,*p*, $f(\theta )=f(\theta_{j})f(\theta_{-j})$, where $f(\theta_{j})$ is the prior used by FCS.

Hughes et al.^20^ show that these conditions are satisfied when the conditional models are normal linear regression models, with the corresponding joint model being the multivariate normal, and the standard noninformative priors are used. Separately, Liu et al.^13^ have given sufficient conditions under which the stationary distribution (assuming it exists) which FCS MI draws from converges (as n→∞) to the posterior distribution of the missing data in a well-defined Bayesian model.

Both Hughes et al.^20^ and Liu et al.^13^ give examples where compatible conditional models are used but FCS is not equivalent to imputation from a Bayesian joint model. A common, and therefore important, example is that in which a binary variable is imputed using logistic regression conditional on a continuous variable, with the latter imputed using a normal linear regression model. Although these models are compatible with each other, FCS imputation fails to utilise the information contained in the marginal distribution of the continuous variable about the logistic regression parameters, and consequently FCS MI does not draw from a Bayesian joint model.

Even when the conditional models are not compatible, Liu et al.^13^ show that provided the conditional models are valid semi-compatible, the estimator ${\overset{\wedge }{\psi }}_{\mathrm{MI}}$ is consistent. This result can be used to conclude that in the linear-logistic example described in the previous paragraph, in which the models are compatible (and therefore also semi-compatible) ${\overset{\wedge }{\psi }}_{\mathrm{MI}}$ is consistent provided both models are correctly specified. Note however that since valid semi-compatibility does not guarantee FCS is equivalent to imputation from a Bayesian joint model, there is no guarantee that Rubin’s rule for the variance will provide valid inferences in this case. If the conditional models used are not even semi-compatible, in general we expect inconsistent estimates.

## 5 FCS imputation accommodating the substantive model

In this section, we describe how the standard FCS algorithm, described in Section 4, can be modified to ensure that each of the univariate imputation models used is compatible with the substantive model. We term the algorithm substantive model compatible FCS (SMC-FCS). To specify an imputation model for *X_j_*, *j* = 1, … , *p* which is compatible with the substantive model, we note that

$$f(X_{j}|X_{-j},Z,Y)=\frac{f(Y,X_{j},X_{-j},Z)}{f(Y,X_{-j},Z)}\propto f(Y|X_{j},X_{-j},Z)f(X_{j}|X_{-j},Z)=f(Y|X,Z)f(X_{j}|X_{-j},Z)$$

In SMC-FCS, for *j* = 1, … , *p*, we specify models $f(X_{j}|X_{-j},Z,\varphi_{j})$, together with noninformative priors $f(\varphi_{j})$. Then, given values of ψ and $\varphi_{j}$, we impute missing values in *X_j_* from the density proportional to

(4) $$f(Y|X,Z,\psi )f(X_{j}|X_{-j},Z,\varphi_{j})$$

In general this density will not belong to a standard parametric family, complicating direct simulation of values. In Section 6, we show how rejection sampling can be used to draw values from it for the most common types of substantive model.

The imputation model for *X_j_* defined by equation (4) depends both on $\varphi_{j}$ and the substantive model parameter ψ. Recall that at the *t*th iteration of the standard FCS algorithm the parameter of the imputation model for *X_j_* is drawn from its posterior conditional on $y,x_{j}^{\mathrm{obs}},x_{-j}^{\left(t\right)},z$. Under the imputation model defined by equation (4), the posterior for $\left(\varphi_{j},\psi \right)$ conditional on $y,x_{j}^{\mathrm{obs}},x_{-j}^{\left(t\right)},z$ is difficult to draw from because subjects with *X_j_* missing contribute $f(Y|X_{-j},Z)$ to this posterior, whose calculation requires *X_j_* to be integrated out from f(Y|X,Z,ψ).

The posterior is however easy to draw from if we condition on (as in a standard Gibbs sampler) $x_{j}^{\mathrm{mis}(t-1)}$ in addition to $y,x_{j}^{\mathrm{obs}},x_{-j}^{\left(t\right)},z$. The posterior is then

$$f(\varphi_{j},\psi |y,x_{j}^{\left(t-1\right)},x_{-j}^{\left(t\right)},z)\propto f(\varphi_{j},\psi )f(y|x_{j}^{\left(t-1\right)},x_{-j}^{\left(t\right)},z,\psi )f(x_{j}^{\left(t-1\right)}|x_{-j}^{\left(t\right)},z,\varphi_{j})$$

Assuming we specify independent priors f(ψ) and $f(\varphi_{j}),f(\varphi_{j},\psi )=f(\varphi_{j})f(\psi )$, and the posterior is proportional to

$$f(\varphi_{j})f(\psi )f(y|x_{j}^{\left(t-1\right)},x_{-j}^{\left(t\right)},z,\psi )f(x_{j}^{\left(t-1\right)}|x_{-j}^{\left(t\right)},z,\varphi_{j})$$

In SMC-FCS the *t*th iteration consists of drawing (up to constants of proportionality)

(5) $$\psi \left(t,j\right)\sim f(\psi )f(y|x_{j}^{\left(t-1\right)},x_{-j}^{\left(t\right)},z,\psi )$$

(6) $$\varphi_{j}^{\left(t\right)}\sim f(\varphi_{j})f(x_{j}^{\left(t-1\right)}|x_{-j}^{\left(t\right)},z,\varphi_{j})$$

and then drawing the missing values of *X_j_* from the density proportional to (4).

In software implementations of the standard FCS algorithm, 10 iterations are typically used to ‘burn-in’, based on empirical experience suggesting this is often sufficient for convergence of the sampler. Since when imputing missing values in *X_j_* SMC-FCS conditions on $x_{j}^{\mathrm{mis}(t-1)}$ (unlike standard FCS), SMC-FCS might be expected to require a larger number of iterations in order to converge to the required stationary distribution, assuming it exists. As with standard FCS, convergence can be assessed by examining plots of means of partially observed variables by iteration number, and also by examining the relative magnitude of between-chain variation and within-chain variation.

### 5.1 Statistical properties

In the appendix, we show that under certain conditions (essentially identical to those given by Hughes et al.^20^ for standard FCS), the SMC-FCS algorithm, at convergence, generates imputations from a Bayesian joint model in which the model for Y|X,Z is the same as the substantive model f(Y|X,Z,ψ). These conditions are as follows:

1. The covariate models $f(X_{j}|X_{-j},Z,\varphi_{j}),j=1,\ldots ,p$, are mutually compatible, with corresponding joint model f(X|Z,φ).
2. For each j=1,…,p, this joint model can be factorised as $f(X|Z,\varphi )=f(X_{j}|X_{-j},Z,\varphi_{j})f(X_{-j}|Z,\varphi_{-j})$, where $\left(\varphi_{j},\varphi_{-j}\right)$ is a reparameterisation of φ, and $\varphi_{j}$ and $\varphi_{-j}$ are variationally independent.
3. There exists a prior f(φ) such that for each $j=1,\ldots ,p,f(\varphi )=f(\varphi_{j})f(\varphi_{-j})$, where $f(\varphi_{j})$ is the prior used by SMC-FCS.

As stated in Section 4.2, Hughes et al.^20^ have shown that these conditions are satisfied when (in our setting) the covariate models are normal linear regressions and the usual noninformative priors are used. When SMC-FCS is equivalent to imputation from a Bayesian joint model, and this model is correctly specified, we expect Rubin’s rules estimator ${\overset{\wedge }{\psi }}_{\mathrm{MI}}$ to be consistent and for the CIs to have nominal coverage.

Following the results of Liu et al.^13^ for standard FCS, if the covariate models $f(X_{j}|X_{-j},Z,\varphi_{j})$ are valid semi-compatible, we conjecture that the MI estimator of the substantive model parameters ${\overset{\wedge }{\psi }}_{\mathrm{MI}}$ will still be consistent. Just as with FCS, however, there is no guarantee however that CIs based on Rubin’s variance estimator will give at least nominal coverage. Lastly, if the covariate models are not valid semi-compatible, in general, we would not expect the estimator ${\overset{\wedge }{\psi }}_{\mathrm{MI}}$ to be consistent.

## 6 Sampling from the imputation model

In this section, we give details of how the method of rejection sampling can be used to sample from the density given in equation (4) for some of the most common types of substantive model. Rejection sampling involves creating draws from a proposal density (from which it is easy to draw), until a draw is made satisfying a particular condition. We choose $f(X_{j}|X_{-j},Z,\varphi_{j})$ as our proposal density, on the assumption that it is easy to sample from this density. To use rejection sampling, the ratio of the target density to the proposal density (up to a constant of proportionality) must be bounded above by a quantity not involving *X_j_*.^21^ Here this ratio is proportional to

(7) $$\frac{f(Y|X,Z,\psi )f(X_{j}|X_{-j},Z,\varphi_{j})}{f(X_{j}|X_{-j},Z,\varphi_{j})}=f(Y|X_{j},X_{-j},Z,\psi )$$

Let $c(Y,X_{-j},Z,\psi )$ denote an upper bound (in *X_j_*) for $f(Y|X_{j},X_{-j},Z,\psi )$. To generate a draw from the density proportional to equation (4), we sample pairs of values $X_{j}^{*}$ from the density given by $f(X_{j}|X_{-j},Z,\varphi_{j})$ and *U* from the uniform distribution on (0,1) until

(8) $$U\leq \frac{f(Y|X_{j}^{*},X_{-j},Z,\psi )}{c(Y,X_{-j},Z,\psi )}$$

When this inequality is satisfied, the value $X_{j}^{*}$ is a draw from the density proportional to equation (4).

We must therefore bound $f(Y|X_{j},X_{-j},Z,\psi )$ by a quantity not involving *X_j_*. The bound will depend on the specification of the substantive model. In the following sections, we derive bounds for the cases of (i) a normal regression model, (ii) a model for a discrete outcome *Y* and (iii) a proportional hazards survival model.

### 6.1 Normal regression

Suppose that the substantive model specifies that *Y* is normal, with conditional mean $E(Y|X)=g(X_{j},X_{-j},Z,\beta )$ for some function *g*(), and residual variance $\sigma_{\varepsilon }^{2}$, so that $\psi =(\beta ,\sigma_{\varepsilon }^{2})$. Then

$$f(Y|X_{j},X_{-j},Z,\psi )=\frac{1}{\sqrt{2\pi \sigma_{\varepsilon }^{2}}}\exp(-(Y-g(X_{j},X_{-j},Z,\beta ))2/2\sigma_{\varepsilon }^{2})\;\leq \frac{1}{\sqrt{2\pi \sigma_{\varepsilon }^{2}}}$$

To generate a draw for the missing value *X_j_*, we draw a value $X_{j}^{*}$ from $f(X_{j}|X_{-j},Z,\varphi_{j})$, and *U* from the uniform distribution on (0, 1). The draw $X_{j}^{*}$ is accepted if

(9) $$U\leq f(Y|X_{j}^{*},X_{-j},Z,\psi )\sqrt{2\pi \sigma_{\varepsilon }^{2}}=\exp(-(Y-g(X_{j}^{*},X_{-j},Z,\beta ))2/2\sigma_{\varepsilon }^{2})$$

If the draw is not accepted, new draws of $X_{j}^{*}$ and *U* are made until they satisfy the condition in equation (9).

### 6.2 Discrete outcomes

Now consider a discrete outcome *Y*. This includes the case of a binary outcome *Y*, which is commonly modelled using logistic regression. When *Y* is discrete, $f(Y|X_{j},X_{-j},Z,\psi )$ is a probability, and hence is less than or equal to one. The rejection sampling algorithm then consists of drawing $X_{j}^{*}$ from $f(X_{j}|X_{-j},Z,\varphi_{j})$ and U~U(0,1), and accepting $X_{j}^{*}$ when

$$U\leq f(Y|X_{j}^{*},X_{-j},Z,\psi )$$

### 6.3 Proportional hazards models

Lastly, suppose that interest lies in the time *T* to an event of interest, but this time may be censored. Let *C* denote the censoring time. We observe *W* = min(*T*, *C*), and D=1(T<C), which denotes whether the subject’s event time has been observed or was censored. We assume that censoring is noninformative, in the sense that T⊥C|X,Z. Furthermore, we assume C⊥X|Z. Together these assumptions allow us to avoid modelling the censoring process.^22^

We assume that the substantive model is the proportional hazards model:

(10) $$h(t|X)=h_{0}(t)\exp(g(X_{j},X_{-j},Z,\beta ))$$

where h(t|X) denotes the hazard at time *t*, *h*_0_(*t*) denotes the baseline hazard at time *t* and $g(X_{j},X_{-j},Z,\beta )$ denotes a function of *X* and *Z* indexed by parameter *β*. In parametric proportional hazards models the baseline hazard function is parametrised by a finite set of parameters λ, so that ψ=(β,λ). In Cox’s proportional hazards model the baseline hazard is allowed to be arbitrary, so that $\psi =(\beta ,h_{0}(\cdot ))$ with $h_{0}(\cdot )$ an infinite-dimensional parameter. Equivalently, we can parametrise the model using the baseline cumulative hazard $H_{0}(t)=\int_{0}^{t}h_{0}(s)\mathrm{ds}$, so that $\psi =(\beta ,H_{0}(\cdot ))$.

We first consider how to sample *X_j_* for a subject for whom *D* = 0, that is, their time to event is censored. Then since by assumption T⊥C|X,Z and C⊥X|Z

$$f(W=t,D=0|X_{j},X_{-j},Z,\psi )=f(T>t,C=t|X_{j},X_{-j},Z,\psi )=P(T>t|X_{j},X_{-j},Z,\psi )f(C=t|X_{j},X_{-j},Z)=P(T>t|X_{j},X_{-j},Z,\psi )f(C=t|Z)\leq f(C=t|Z)$$

We draw $X_{j}^{*}$ from $f(X_{j}|X_{-j},Z,\varphi_{j})$ and U ~U(0,1), and accept $X_{j}^{*}$ when

$$U\leq \frac{f(W=t,D=0|X_{j}^{*},X_{-j},Z,\psi )}{f(C=t|Z)}=P(T>t|X_{j}^{*},X_{-j},Z,\psi )=\exp(-H_{0}(t)eg(X_{j}^{*},X_{-j},Z,\beta ))$$

For a subject who is not censored (*D* = 1), we have

$$f(W=t,D=1|X_{j},X_{-j},Z,\psi )=P(C>t|X_{j},X_{-j},Z)h(t|X_{j},X_{-j},Z,\psi )P(T>t|X_{j},X_{-j},Z,\psi )=P(C>t|Z)h_{0}(t)\exp(g(X_{j},X_{-j},Z,\beta )-H_{0}(t)eg(X_{j},X_{-j},Z,\beta ))$$

Since exp() is monotonically increasing, this expression takes its maximum when $g(X_{j},X_{-j},\beta )-H_{0}(t)eg(X_{j},X_{-j},\beta )$ takes its maximum. Differentiating this with respect to *g*() and setting the resulting expression to zero shows that this occurs when $H_{0}(t)eg(X_{j},X_{-j},\beta )=1$. Therefore

$$f(W=t,D=1|X_{j},X_{-j},Z,\psi )\leq P(C>t|Z)\frac{h_{0}(t)e-1}{H_{0}(t)}$$

We can thus draw $X_{j}^{*}$ from $f(X_{j}|X_{-j},Z,\varphi_{j})$ and U~U(0,1), and accept $X_{j}^{*}$ when

$$U\leq \frac{f(W=t,D=1|X_{j}^{*},X_{-j},Z,\psi )}{P(C>t|Z)\frac{h_{0}(t)e-1}{H_{0}(t)}}=\exp(1+g(X_{j}^{*},X_{-j},Z,\beta )-H_{0}(t)eg(X_{j}^{*},X_{-j},Z,\beta ))H_{0}(t)$$

## 7 Simulation study

In this section, we describe the results of simulation studies to investigate the performance SMC-FCS in situations in which the substantive model is incompatible with standard choices for covariate imputation models.

### 7.1 Linear regression with quadratic covariate effects

We first simulated from a linear regression substantive model with a single covariate *X* with linear and quadratic effects, for which standard imputation model choices for the covariate *X* are incompatible.

#### 7.1.1 Simulation setup

The outcome *Y* was simulated according to

$$Y=\beta_{0}+\beta_{1}X+\beta_{2}X2+\varepsilon$$

with *β*_0_ = 4, $\beta_{1}=-4$, *β*_2_ = 1 and $\varepsilon \sim \text{iid}N(0,\sigma_{\varepsilon }^{2})$. These coefficients were chosen to give a moderately strong U-shaped association between *Y* and *X*. The variance $\sigma_{\varepsilon }^{2}$ was chosen such that the coefficient of determination *R*^2^ was equal to 0.5.

The covariate *X* was simulated from a normal, a log-normal or a normal mixture distribution. For all three distributions *X* had mean 2 and variance 1. For the log-normal distribution, *X* was generated by exponentiating a draw from $N(\log(\sqrt{3.2}),\log(5/4))$. For the normal mixture distribution, *X* was drawn from *N*(1.125,0.234) with probability 0.5 and from *N*(2.875,0.234) with probability 0.5.

For each distribution of *X*, values were made missing either according to the missing completely at random (MCAR) mechanism P(R=1|X,Y)=0.7 or the MAR mechanism $P(R=1|X,Y)=\text{expit}(\alpha_{0}+\alpha_{1}Y)$, where expit(a)=(1+exp(-a))-1, $\alpha_{1}=-1/\text{SD}(Y)$ and *α*_0_ was chosen to make the marginal probability of observing *X* equal to 0.7. In all simulations datasets for *n* = 1000 subjects were generated and 1000 simulations were performed for each scenario.

#### 7.1.2 Estimation methods

For each simulated dataset we first imputed the missing values of *X* using a linear regression model with the ice command in Stata. We used the default imputation model, with the expectation of *X* modelled as a linear function of *Y*. Note that since here there is only one partially observed variable, no iteration is required within FCS. Missing values of *X*^2^ were then passively imputed as the square of these imputed values of *X* (‘linear passive’). Second, we imputed the missing *X* values using the ‘transform then impute’ or ‘just another variable’ (JAV) approach proposed by von Hippel,^15^ that is, by treating *X*^2^ as another variable to be imputed in the ice command in Stata. Third, we imputed *X* using the mice.impute.quadratic function in the R MICE package (‘polynomial combination’). This implements a method recently proposed by van Buuren^23^ (p. 140), which imputes the linear combination of *X* and *X*^2^ which enters in the linear predictor of the substantive model, followed by solving a quadratic equation for *X*. Lastly, we used SMC-FCS, assuming *X* is marginally normally distributed for all scenarios. We chose to implement SMC-FCS using the same marginal model for *X* to explore the performance of (substantive model) compatible but mis-specified imputation models. For all imputation approaches, 10 imputed datasets were generated, and estimates and CIs found using Rubin’s rules. We used 10 iterations per imputation in SMC-FCS and the default 10 iterations in the ice command. With *X* univariate, SMC-FCS is equivalent to imputation from the corresponding Bayesian model. We used standard noninformative priors for normal linear regression parameters in SMC-FCS, that is, f(β,σ2)∝1/σ2, where *β* and *σ*^2^ denote the vector of regression coefficients and residual variance, respectively.

#### 7.1.3 Results

Table 2 shows the results of the simulations, showing the empirical mean and standard deviation of estimates of *β*_2_ and the coverage of nominal 95% CIs. With normally distributed *X* and MCAR, linear passive imputation resulted in biased estimates, with considerable attenuation in ${\overset{\wedge }{\beta }}_{2}$ towards zero as expected. Here the imputation model being used is incompatible (with the substantive model) and mis-specified. CI coverage for linear passive imputation was also extremely poor, with zero coverage for *β*_2_. JAV, polynomial combination and SMC-FCS gave unbiased results, with similar efficiency to each other. SMC-FCS had CI coverage close to 95%, but JAV and polynomial combination had slightly low coverage. With *X* log-normally distributed and MCAR linear passive imputation was again biased with poor CI coverage. JAV was unbiased, although estimates were considerably more variable SMC-FCS. Furthermore, the coverage of the CI for *β*_2_ from JAV was only 83%. The polynomial combination method performed similar to JAV here. Despite the assumed model for *X* being mis-specified, SMC-FCS was unbiased and the 95% CI for *β*_2_ had the correct coverage. With *X* distributed according to a normal mixture model and MCAR, JAV and polynomial combination were again unbiased. The CI coverage of JAV and polynomial combination for *β*_2_ was close to 95%. Linear passive imputation continued to be severely biased. SMC-FCS was somewhat biased towards the null for *β*_2_, and consequently CI coverage for *β*_2_ was only 66%.

**Table 2.** Simulation results – linear regression with quadratic covariate effects. Empirical mean (SD) of estimates of quadratic coefficient *β*_2_ = 1 from 1000 simulations, using linear passive imputation, JAV imputation, the polynomial combination method and SMC-FCS. Empirical coverage of nominal 95% confidence intervals is also shown (Cov). Monte-Carlo errors for means and SDs are less than 0.003, except for log-normal *X* MAR, where Monte-Carlo errors for means and SDs are less than 0.02. Monte-Carlo errors for confidence interval coverage are less than 1.6%.

Scenario	Linear passive		JAV		Polynomial comb.		SMC-FCS	
	Mean (SD)	Cov	Mean (SD)	Cov	Mean (SD)	Cov	Mean (SD)	Cov
*X* MCAR
Normal *X*	0.696 (0.041)	0.0	1.001 (0.041)	91.9	1.005 (0.040)	91.5	0.998 (0.038)	93.9
Log-normal *X*	0.789 (0.084)	16.7	1.012 (0.100)	82.6	1.025 (0.097)	83.1	1.000 (0.059)	96.2
*X* mixture of normals	0.493 (0.036)	0.0	0.997 (0.036)	94.7	1.003 (0.034)	95.8	0.942 (0.036)	65.9
*X* MAR
Normal *X*	0.618 (0.045)	0.0	1.192 (0.073)	12.3	1.045 (0.069)	75.9	0.995 (0.049)	94.4
Log-normal *X*	0.790 (0.265)	58.6	1.488 (0.324)	25.7	1.288 (0.179)	27.6	1.002 (0.158)	91.7
*X* mixture of normals	0.450 (0.033)	0.0	1.085 (0.047)	48.1	1.009 (0.048)	87.8	0.840 (0.038)	3.4

JAV: just another variable; MCAR: missing completely at random; MAR: missing at random.

With normal *X* and MAR, linear passive imputation gave biased estimates and the CI for *β*_2_ had zero coverage. With data MAR, JAV no longer gave unbiased estimates, in agreement with the findings of Seaman et al.,^10^ and the CI for *β*_2_ had only 12% coverage. Polynomial combination had only slight bias, but CI coverage for *β*_2_ was only 75.9%. In contrast, SMC-FCS was unbiased and the CI for *β*_2_ had approximately 95% coverage. All estimators were considerably more variable with *X* log-normal MAR. JAV and polynomial combination had considerable bias and poor CI coverage for β_2_, as did linear passive imputation. Despite using a mis-specified model for the distribution of *X*, SMC-FCS was again unbiased, although the CI for β_2_ had slightly lower than nominal coverage. Lastly with *X* distributed as a mixture of two normals and MAR, linear passive imputation continued to be substantially biased. JAV was biased to a lesser extent, although its CI for *β*_2_ had poor coverage. SMC-FCS was biased (since the assumed distribution for *X* was incorrect) towards zero, and its CI for β_2_ had extremely poor coverage, due to the estimator’s large bias relative to its variability. The polynomial combination method performed best here, with unbiased estimates of β_2_ and only somewhat reduced CI coverage.

### 7.2 Linear regression with interaction

Next, we considered a linear regression substantive model in which two covariates interact with each other in their effect on outcome.

#### 7.2.1 Simulation setup

The outcome *Y* was generated according to

$$Y=\beta_{0}+\beta_{1}X_{1}+\beta_{2}X_{2}+\beta_{3}X_{1}X_{2}+\varepsilon$$

with *β*_0_ = 0, *β*_1_ = 1, *β*_2_ = 1, *β*_3_ = 1, and with $\varepsilon \sim \text{iid}N(0,\sigma_{\varepsilon }^{2})$, where as before, $\sigma_{\varepsilon }^{2}$ was chosen to give *R*^2 ^= 0.5.

In the first scenario, *X*_1_ and *X*_2_ were generated from a bivariate normal distribution, with each covariate having mean 2 and variance 1, and the correlation between the two equal to 0.5. To explore robustness of the imputation methods to violations of normality assumptions, in a second scenario $\log(X_{1})$ and $\log(X_{2})$ were generated from a bivariate normal distribution so that they both had marginal distribution $N(\log(\sqrt{3.2}),\log5/4)$ and the correlation between the two was equal to 0.5. To investigate robustness to linearity assumptions between covariates, in a third scenario we generated $X_{1}\sim N(2,1)$ and $X_{2}|X_{1}\sim N((X_{1}-2)2,2)$. Fourth, we generated *X*_1_ from a Bernoulli distribution with probability 0.5, and $X_{2}|X_{1}\sim N(X_{1},1)$. To explore robustness to violations of normality assumptions, in the final scenario we generated *X*_1_ from a Bernoulli distribution with probability 0.5 and $X_{2}=X_{1}+\exp(v)$ where $v\sim N(\log(\sqrt{3.2}),\log(5/4))$.

Values in both *X*_1_ and *X*_2_ were first made (independently) MCAR, each with probability 0.7 of being observed. We then repeated the simulations with *X*_1_ observed with probability $\text{expit}(\alpha_{0}+\alpha_{1}Y)$ where $\alpha_{1}=-1/\text{SD}(Y)$ and *α*_0_ was chosen to make the marginal probability of observing *X*_1_ equal to 0.7, and with *X*_2_ also observed with probability $\text{expit}(\alpha_{0}+\alpha_{1}Y)$.

#### 7.2.2 Estimation methods

For each simulated dataset we first imputed using the ice command in Stata, followed by passive imputation of the interaction term (‘passive FCS’). A linear regression imputation model was used when the covariate was continuous and a logistic regression imputation model when the covariate was binary. In the imputation model for *X*_1_ (*X*_2_) the outcome *Y*, *X*_2_ (*X*_1_) and their interaction *YX*_2_ (*YX*_1_) were included as explanatory variables.

We obtained estimates using JAV by including the interaction variable *X*_1_*X*_2_ as an additional variable in the ice command. Covariate *X*_1_ (*X*_2_) was imputed using a linear regression model (even when *X*_1_ was binary) with *Y*, *X*_2_ (*X*_1_) and *X*_1_*X*_2_ as explanatory variables. The interaction term *X*_1_*X*_2_ was imputed using a linear regression model with *Y*, *X*_1_ and *X*_2_ as explanatory variables. Since all conditional imputation models are linear regressions with other variables included linearly, JAV is equivalent here to imputation from a trivariate normal imputation model for (*X*_1_,*X*_2_,*X*_1_*X*_2_) conditional on *Y*.

Lastly, we obtained estimates using SMC-FCS, assuming a normal regression model for $X_{1}|X_{2}$ or a logistic regression model when *X*_1_ was binary. A linear regression model was assumed for $X_{2}|X_{1}$. When assuming $X_{1}|X_{2}$ and $X_{2}|X_{1}$ are linear regressions, SMC-FCS is equivalent to imputation from the Bayesian model defined by the substantive model and a bivariate normal model for (*X*_1_,*X*_2_). In contrast, when assuming a logistic regression model for $X_{1}|X_{2}$ and a linear regression for $X_{2}|X_{1}$, although these models are compatible, SMC-FCS is not equivalent to imputation from a Bayesian model. When drawing from the posterior of the logistic regression parameters (equation (5)) we used a multivariate normal, with mean equal to the MLE and variance covariance corresponding to the inverse of the ‘observed’ data information matrix.

#### 7.2.3 Results

Table 3 shows the mean (SD) estimates of *β*_1_ and *β*_3_ and empirical coverage of the corresponding 95% CIs. With data MCAR and *X*_1_ and *X*_2_ bivariate normal, passive FCS was substantially biased and had poor CI coverage for *β*_1_ and *β*_3_. In contrast, both JAV and SMC-FCS were unbiased. However, SMC-FCS was somewhat more efficient than JAV. CI coverage for β_3_ was at the nominal level for both JAV and SMC-FCS. With *X*_1_ and *X*_2_ distributed log-normal, passive FCS had slightly larger bias for β_1_ and β_3_ and again poor CI coverage. JAV continued to be unbiased with correct CI coverage. SMC-FCS was somewhat biased, due to mis-specification of the models for $X_{1}|X_{2}$ and $X_{2}|X_{1}$, although CI coverage was only slightly below the nominal level for *β*_1_ and *β*_3_. When *X*_2_ was normally distributed with mean a quadratic in *X*_1_, passive FCS was again biased. JAV continued to be approximately unbiased. SMC-FCS was again somewhat biased, with CI coverage for *β*_3_ approximately 88%. With *X*_1_ Bernoulli and $X_{2}|X_{1}$ normal, both JAV and SMC-FCS were unbiased, although SMC-FCS was slightly more efficient. Both had empirical CI coverage of approximately 95% for both *β*_1_ and *β*_3_. It is important to note that here SMC-FCS is not equivalent to imputation a Bayesian model, and thus there is no guarantee that Rubin’s rules will give asymptotically unbiased variance estimates. That the CIs from SMC-FCS had the correct coverage in this setting is thus encouraging. Passive FCS was again biased. As expected, with *X*_2_ log-normal given *X*_1_ JAV continued to remain approximately unbiased while SMC-FCS had moderately large biases for β_1_ and β_3_, although CI coverage was only slightly below 95%.

**Table 3.** Simulation results – linear regression with interaction. Empirical mean (SD) of estimates of *β*_1_ = 1 and *β*_3_ = 1 from 1000 simulations, standard FCS with passive imputation (Passive FCS), JAV imputation and SMC-FCS. Empirical coverage of nominal 95% confidence intervals is also shown (Cov). Monte-Carlo errors for means and SDs are all less than 0.04 for *β*_1_ and less than 0.02 for *β*_3_. Monte-Carlo errors for confidence interval coverage are less than 1.6%.

Passive FCS		JAV		SMC-FCS	
*X*_1_,*X*_2_ distribution	Parameter	Mean (SD)	Cov	Mean (SD)	Cov	Mean (SD)	Cov
MCAR
*X*_1_,*X*_2_ bivariate normal	β_1_	1.50 (0.39)	82.7	1.02 (0.53)	94.5	0.99 (0.45)	95.4
β_3_	0.74 (0.14)	75.1	1.00 (0.23)	95.2	1.01 (0.19)	95.4
*X*_1_,*X*_2_ bivariate log-normal	β_1_	1.70 (0.57)	72.4	1.03 (0.61)	95.1	0.81 (0.56)	92.7
β_3_	0.69 (0.23)	64.5	1.00 (0.23)	94.8	1.08 (0.22)	92.1
*X*_1_ normal, $X_{2}|X_{1}\sim N((X_{1}-2)2,2)$	β_1_	2.20 (0.65)	36.6	1.03 (0.50)	95.3	1.09 (0.52)	93.0
β_3_	0.70 (0.27)	57.6	1.00 (0.13)	94.6	1.09 (0.14)	87.9
*X*_1_ Bernoulli, $X_{2}|X_{1}$ normal	β_1_	1.11 (0.21)	91.8	1.00 (0.23)	95.3	0.99 (0.22)	95.0
β_3_	0.80 (0.15)	80.7	0.99 (0.20)	95.3	0.99 (0.17)	94.6
*X*_1_ Bernoulli, $X_{2}|X_{1}$ log-normal*	β_1_	1.74 (0.63)	78.3	1.01 (0.74)	95.1	1.28 (0.65)	92.1
β_3_	0.71 (0.24)	78.4	0.98 (0.28)	94.5	0.89 (0.24)	92.0
MAR
*X*_1_,*X*_2_ bivariate normal	β_1_	1.63 (0.37)	78.6	1.31 (0.60)	91.1	1.03 (0.46)	95.5
β_3_	0.64 (0.12)	56.2	0.96 (0.30)	94.0	0.97 (0.19)	95.3
*X*_1_,*X*_2_ bivariate log-normal*	β_1_	2.53 (0.95)	45.5	1.57 (1.17)	91.7	1.02 (0.93)	94.9
β_3_	0.16 (0.35)	30.0	1.06 (0.57)	95.0	1.00 (0.45)	92.2
*X*_1_ normal, $X_{2}|X_{1}\sim N((X_{1}-2)2,2)$**	β_1_	2.39 (1.33)	41.9	1.68 (0.63)	81.1	1.29 (0.54)	94.8
β_3_	0.13 (0.28)	24.3	1.17 (0.20)	84.3	1.10 (0.20)	90.7
*X*_1_ Bernoulli, $X_{2}|X_{1}$ normal	β_1_	1.11 (0.21)	92.2	1.14 (0.22)	88.4	1.00 (0.22)	95.0
β_3_	0.78 (0.15)	81.6	0.97 (0.22)	95.3	0.98 (0.17)	95.6
*X*_1_ Bernoulli, $X_{2}|X_{1}$ log-normal	β_1_	1.84 (0.74)	85.1	1.11 (0.99)	95.1	1.24 (0.77)	93.1
β_3_	0.68 (0.28)	84.6	0.96 (0.38)	94.8	0.91 (0.28)	92.9
Results based on * 999, ** 968 simulations

FCS: fully conditional specification; JAV: just another variable; SMC-FCS: substantive model compatible-fully conditional specification; MCAR: missing completely at random; MAR: missing at random.

When *X*_1_ and *X*_2_ were MAR passive FCS continued to be biased for all *X*_1_,*X*_2_ distributions considered. With *X*_1_,*X*_2_ bivariate normal, estimates from JAV had a small bias towards zero for β_3_, but a larger bias for β_1_. In contrast, SMC-FCS was unbiased and more efficient. In a number of simulated datasets with covariates log-normally distributed or with *X*_2_ quadratic given *X*_1_ the FCS algorithm created imputed datasets with extremely large imputed values of *X*_1_ and *X*_2_, resulting in a co-linearity error when attempting to fit the substantive model to the imputations. Consequently, for these scenarios results are shown for the subset of the simulated datasets for which estimates from all methods were obtained. JAV was approximately unbiased for β_3_ when the covariates were log-normally distributed, but was substantially biased for *β*_1_. SMC-FCS was approximately unbiased here. With *X*_2_ given *X*_1_ normal with mean quadratic in *X*_1_, both JAV and SMC-FCS were biased, but again biases for *β*_1_ and *β*_3_ were smaller for SMC-FCS. Lastly, with *X*_1_ binary and *X*_2_ either conditionally normal or log-normal, JAV had little bias for *β*_3_, but had some bias for *β*_1_. SMC-FCS was unbiased for both *β*_1_ and *β*_3_ when *X*_2_ was conditionally normal given *X*_1_. With *X*_2_ log-normally distributed, both JAV and SMC-FCS were somewhat biased for *β*_1_ and *β*_3_, although the biases of JAV were smaller.

### 7.3 Cox proportional hazards models

Lastly, we performed simulations for imputing missing covariates with a Cox proportional hazards model. Recently, White and Royston^16^ derived approximate results to inform the choice of imputation model in this context. They recommended including the event indicator and the Nelson–Aalen estimate of the marginal cumulative hazard function as covariates in imputation models. Their simulation results showed that this approach generally worked well for imputing normally distributed covariates, except when the covariate effects were large, when some attenuation towards the null occurred.

#### 7.3.1 Simulation setup

Survival times were simulated with hazard function $h(t|X)=0.002\exp(\beta_{1}X_{1}+\beta_{2}X_{2})$ with $\beta =(\beta_{1},\beta_{2})=(1,1)$. Censoring times were generated from an exponential distribution with hazard 0.002. We simulated *X*_1_ from a Bernoulli distribution with probability 0.5, and $X_{2}|X_{1}\sim N(X_{1},1)$. Values in *X*_1_ and *X*_2_ were made (independently) MCAR, with probability of observation 0.7. We performed simulations with *n* = 1000 subjects and also with *n* = 100 subjects.

#### 7.3.2 Estimation methods

For each simulated dataset we first estimated *β* by fitting the Cox proportional hazards model to the complete cases (CC). Next, we multiply imputed the missing values in *X*_1_ and *X*_2_ using FCS (10 imputations). A linear regression imputation model was used for *X*_2_ and a logistic regression model for *X*_1_. Following the recommendations of White and Royston,^16^ we included the event indicator *D* and the Nelson–Aalen estimate of the marginal cumulative hazard as covariates in both imputation models (FCS). Lastly, we estimated *β* using SMC-FCS as described in Section 6.3, assuming a logistic regression model for $X_{1}|X_{2}$ and a linear regression model for $X_{2}|X_{1}$. As described previously, the SMC-FCS algorithm involves taking draws from the posterior distribution of the parameter ψ in the substantive model. For Cox’s proportional hazards model $\psi =(\beta ,H_{0}(\cdot ))$, where *H*_0_(·) is an infinite-dimensional parameter representing the arbitrary baseline hazard function. It is unclear how a draw can be made from the posterior distribution of *H*_0_(·), and indeed whether Rubin’s rules can be expected to give asymptotically unbiased variance estimates in a semi-parametric model. In our simulation study we allowed for uncertainty in *β* by drawing a new value from a (bivariate) normal distribution with mean equal to the current estimate of *β* and with covariance matrix based on the usual ‘observed’ data information matrix. We then updated *H*_0_(·) using the usual Breslow estimator, conditioning on the newly drawn value of *β*.

#### 7.3.3 Results

Table 4 shows the results from the 1000 simulations. CC is consistent here, since missingness is completely at random. However, with *n* = 100 CC showed some upward finite sample bias for both *β*_1_ and *β*_2_. In accordance with the results of White and Royston,^16^ FCS resulted in somewhat biased estimates, with the bias larger for the coefficient corresponding to the continuous covariate, although CI coverage for both *β*_1_ and *β*_2_ was approximately 95%. SMC-FCS, like CC, showed some slight upward bias, but was somewhat more efficient. Of interest was that the CIs had correct coverage, despite the fact that our implementation ignores uncertainty in the baseline hazard function.

**Table 4.** Cox proportional hazards outcome model simulation results. Empirical mean (SD) of estimates of β_1_ = 1 and β_2_ = 1 from 1000 simulations, using complete case analysis, MI of *X*_1_ and *X*_2_ using FCS with the event indicator and Nelson–Aalen marginal baseline cumulative hazard function as covariates (FCS), and SMC-FCS. Empirical coverage of nominal 95% confidence intervals is also shown (Cov). Monte-Carlo errors in means and SDs are no more than 0.02 for *n* = 100 and 0.005 for *n* = 1000.

Parameter	Complete case		FCS		SMC-FCS	
	Mean (SD)	Cov	Mean (SD)	Cov	Mean (SD)	Cov
*n* = 100
β_1_ = 1	1.04 (0.47)	95.6	0.94 (0.36)	96.5	1.02 (0.41)	94.7
β_2_ = 1	1.05 (0.26)	95.6	0.89 (0.17)	94.0	1.05 (0.21)	94.8
*n* = 1,000
β_1_ = 1	1.000 (0.129)	95.2	0.902 (0.107)	89.1	1.002 (0.114)	95.0
β_2_ = 1	1.007 (0.070)	94.8	0.861 (0.049)	45.7	1.006 (0.058)	95.1

FCS: fully conditional specification; SMC-FCS: substantive model compatible-fully conditional specification.

For *n* = 1000, CC was essentially unbiased. The biases of FCS were larger than for *n* = 100, which is due to the fact that the finite sample bias, which acted in the opposite direction to the bias caused by the approximation used in the FCS approach, had largely disappeared. Consequently, CI coverage was below the nominal 95% level, with coverage for β_2_ particularly poor at 47%. In contrast, SMC-FCS was unbiased and had correct CI coverage.

## 8 Analysis of data from ADNI

Table 5 shows the estimated log hazard ratios from the substantive model fitted to the *n* = 127 complete cases (of whom 61 converted to AD). This showed borderline evidence of an association between CSF A$\beta_{1-42}$ and hazard of conversion, and borderline significant evidence of curvature in the association, in agreement with the findings of Jack et al.^11^ The estimated association suggests that increasing A$\beta_{1-42}$ is associated with increased hazard of conversion up until a value of ≈14 ng/mL, after which hazard decreases. There was evidence that increased levels of p-tau were associated with increased hazard of conversion. Contrary to what we expected, having a mother or father with AD was suggestive of lower hazard of conversion to AD, although neither coefficient was statistically significant. Hippocampal volume was the strongest predictor of hazard (measured by statistical significance), with larger volumes associated with lower hazard of conversion. This is consistent with the findings of previous studies which have found that the hippocampus is one of the earliest structures of the brain to undergo atrophy during AD.

**Table 5.** Estimates of log hazard ratios (standard errors) for Cox proportional hazards model relating hazard of conversion to AD to baseline risk factors. Estimates based on complete case, FCS imputation and SMC-FCS.

(*n* = 127)	(*n* = 382)
Variable	Complete case	FCS	SMC-FCS
A$\beta_{1-42}$ (ng/mL)	0.31 (0.19)	0.08 (0.10)	0.28 (0.16)
A$\beta_{1-42}^{2}$ (ng^2^/mL^2^)	−0.011 (0.005)	−0.004 (0.003)	−0.010 (0.005)
log(tau) (log pg/mL)	−0.60 (0.47)	−0.23 (0.37)	−0.19 (0.36)
log(p-tau) (log pg/mL)	1.29 (0.51)	0.52 (0.38)	0.44 (0.40)
Mother had AD	−0.61 (0.32)	−0.15 (0.22)	−0.14 (0.21)
Father had AD	−1.07 (0.68)	−0.22 (0.35)	−0.28 (0.34)
Intracranial volume (cm^3^)	0.0005 (0.0010)	0.0010 (0.0007)	0.0011 (0.0007)
Hippocampal volume (cm^3^)	−0.64 (0.17)	−0.47 (0.10)	−0.51 (0.10)
APOE4 positive	−0.06 (0.30)	0.31 (0.22)	0.41 (0.20)

FCS: fully conditional specification; SMC–FCS: substantive model compatible-fully conditional specification.

Next, we used FCS MI to impute the partially observed baseline variables. 50 imputations were used. Continuous variables were imputed using linear regression models while binary variables were imputed using logistic regressions. To incorporate the censored time to conversion outcome we followed the recommendations of White and Royston^16^ and included the event indicator and marginal Nelson–Aalen cumulative hazard function as covariates in the imputation models. We passively imputed the A$\beta_{1-42}^{2}$ term in the imputed datasets. The FCS estimate of A$\beta_{1-42}^{2}$ is smaller in magnitude than the CC estimate (Table 5). This is consistent with the simulation results of Section 7.1, which showed that linear imputation of variables for substantive models which include quadratic effects of the variable leads to attenuation in the estimate of curvature. The coefficient for the linear A$\beta_{1-42}$ term is also much smaller and no longer statistically significant. The estimated coefficient for p-tau is much smaller. The negative association between hippocampal volume and hazard of conversion remained. The coefficients for family history of AD changed by a proportionately large amount. Further investigation revealed that the dependence of hazard of conversion to AD on family history of AD varied (in a model without the CSF variables) according to whether or not the CSF variables were measured. This means that the assumption required for validity of complete case analysis failed for the reduced model without the CSF variables, and this is the likely cause of the large change in the coefficients for family history of AD. Standard errors were considerably smaller, consistent with the gain in information through inclusion of subjects with some missing values into the analysis.

Lastly, we imputed using SMC-FCS, again using 50 imputations, with 10 iterations per imputation. Here we assumed linear regression covariate models for partially observed continuous variables and logistic regressions for partially observed binary variables. Comparing the estimates from SMC-FCS with complete case and passive FCS, we see that the linear and quadratic coefficients of A$\beta_{1-42}$ are much closer to the complete case estimates, with the statistical significance of the quadratic coefficient preserved (Table 5). The estimated coefficient for p-tau is similar to that obtained in the FCS analysis. For the other coefficients the SMC-FCS estimates and CIs are similar to those from FCS. In conclusion, consistent with our earlier simulation results, the results of this analysis suggest that ignoring the quadratic association at the imputation stage leads to attenuation in the corresponding coefficient. In contrast, imputation of the partially observed covariates using SMC-FCS preserved a quadratic association between CSF A$\beta_{1-42}$ seen in the complete cases. Furthermore, use of MI has here led to practically important improvements in the precision of estimated associations, compared to CC.

## 9 MI of covariates in practice

Our developments thus far have assumed that at the imputation stage we have a single correctly specified substantive model f(Y|X,Z,ψ). Usually, we will not know in advance of analysing the data what is an appropriate model for the outcome *Y* of interest given the covariates. One of the apparent advantages of using MI is that once a set of imputed datasets have been generated, a number of different substantive models can be fitted and compared. It is important to note that the validity of estimates from different models fitted to a set of MIs depends on whether the imputation model is correctly specified. In practice, all imputation models are likely to be mis-specified to some extent, but biases may be small provided imputation models preserve those features of the data which are subsequently investigated. It is arguably therefore unrealistic in practice to expect a single set of multiply imputed datasets to be suitable for all possible subsequent types of analysis.

Performing model selection in combination with MI is a challenging problem generally,^24^ and therefore deciding on what substantive model to specify when using the SMC-FCS algorithm is not a trivial process. One possible approach would be to use the complete cases to select the substantive model. Alternatively, the SMC-FCS algorithm could be used to impute the partially observed covariates assuming a rich model for f(Y|X,Z,ψ) which contains as special cases various simpler models that one may wish to fit to the imputed datasets. This approach would mean the imputation models used would be compatible with this larger model, and semi-compatible with those substantive models nested within it. This advice follows that given by others (e.g. Meng^6^ and Schafer^7^) for application of MI in general, whereby imputation models are used which are rich and do not impose assumptions which are subsequently to be relaxed in substantive models. For example, if one believes that two covariates may interact in their effect on *Y*, one could impute compatibly with a model f(Y|X,Z,ψ) which includes the corresponding interaction.

We do not consider the requirement to specify a substantive model at the imputation stage to be a shortcoming of the SMC-FCS approach, since the issue of compatibility between imputation and substantive models is similarly present when one imputes using standard FCS MI. Indeed, whereas SMC-FCS forces the analyst to consider the issue of compatibility between these models, if using standard FCS MI one may unwittingly specify covariate imputation models which are incompatible with one’s substantive model.

As noted in Section 1, in many settings auxiliary variables *V* may be available, which although not involved in the substantive model, may be useful for inclusion in imputation models in order to improve efficiency (by virtue of their association with variables being imputed) or to increase the plausibility of the MAR assumption. The notion of compatibility between imputation and substantive models does not then apply, since the two models involve different sets of variables. However, one could include the auxiliary variables *V* as additional covariates in the model for *Y* in the SMC-FCS algorithm, following which models for *Y* can be fitted using the imputed datasets which omit *V*.

## 10 Discussion

MI is an attractive approach for handling missingness in covariates of regression models. When the substantive model contains non-linearities or interactions, existing imputation approaches using the FCS algorithm may give biased estimates because the imputation models are incompatible with the substantive model. Our proposed modification of the popular FCS approach to MI ensures that each covariate is imputed from a model which is compatible with the substantive model. Although compatibility does not guarantee the imputation models are correctly specified, it ensures that the imputation and substantive models do not make conflicting assumptions which may induce bias in parameter estimates. In our simulations across a range of settings we found SMC-FCS to generally outperform existing alternative imputation approaches.

In special cases, SMC-FCS is equivalent to imputation from a Bayesian joint model, and thus inherits the latter’s statistical properties. More generally, if the covariate models used in SMC-FCS are valid semi-compatible, we conjecture that the resulting estimator is consistent, which is supported by our simulation results. Further, in these cases CI coverage for estimates from SMC-FCS attained nominal coverage, despite the lack of equivalence to imputation from a Bayesian joint model. In simulations in which the covariate models were mis-specified, estimates from SMC-FCS were still less biased than those from what might be considered ‘standard FCS’.

For linear substantive models which contain non-linear covariate effects or interactions, the ‘JAV’ approach is attractive, and is consistent if data are MCAR. This holds irrespective of the joint distribution of the outcome and covariates. However when data are MAR, or for other substantive model types such as logistic regression, we and Seaman et al.^10^ have shown that JAV gives biased estimates. At least in our limited simulation study, the polynomial combination method recently proposed by van Buuren^23^ was superior to JAV, with less bias and coverage closer to the nominal level. A limitation of this approach however is that it only applies to imputation of covariates which have a quadratic association with outcome, and it is unclear whether it can be generalised to substantive models other than linear regression.

Relative to standard FCS MI, SMC-FCS is more computationally intensive because of the use of rejection sampling to sample from the required densities. For example, the SMC-FCS algorithm took six times longer than standard FCS to create 10 imputations for a simulated dataset from the first simulation scenario (linear regression with quadratic covariate effects). The acceptance rate of the rejection sampler will be low when the target density $f(X_{j}|X_{-j},Z,Y)$ differs substantially from the candidate density $f(X_{j}|X_{-j},Z)$. This will occur if a subject has an outcome value *Y* which is unlikely to have occurred given the values of $X_{-j}$ and *Z*. However, our experience thus far in simulation studies has been that this has not been an issue. Furthermore, as for standard FCS MI, additional work is needed to understand the statistical properties of the SMC-FCS algorithm. In some settings, substantive models may be fitted to imputed datasets for a number of different outcomes, and a limitation of our approach is that imputation models are defined with respect to a single (possibly multivariate) outcome variable.

We note that compatibility between imputation and substantive models is closely related to the concept of congeniality defined by Meng.^6^ We chose not to adopt this term because Meng’s definition of congeniality depends additionally on specification of incomplete and complete data ‘procedures’ which give asymptotically equivalent inferences to those under a Bayesian model. Further, in many cases SMC-FCS is not equivalent to imputation from a joint model, and so would not satisfy Meng’s definition of congeniality, which assumes imputation is from a well-defined Bayesian joint model. Lastly, the setup adopted by Meng assumed that covariates are fully observed.

In this article, we have assumed that the outcome is fully observed. In the absence of auxiliary variables subjects with missing outcome provide little or no additional information regarding the substantive model parameters,^25^ such that imputation of missing outcomes may not be beneficial. Nevertheless, the SMC-FCS algorithm can be readily extended to impute missing outcome values by imputing from the assumed substantive model.

A Stata program implementing SMC-FCS for linear, logistic and Cox proportional hazards models of interest is available for free download from www.missingdata.org.uk.

## Appendix

In this appendix, we show that, under certain conditions, at convergence the SMC-FCS algorithm generates imputations from a well-defined Bayesian model in which the model for Y|X,Z is the substantive model f(Y|X,Z,ψ). First, assume that the covariate models $f(X_{j}|X_{-j},Z,\varphi_{j}),\varphi_{j}\in \Phi_{j},j=1,\ldots ,p$, are mutually compatible, with corresponding joint model f(X|Z,φ),φ∈Φ. For each *j* = 1, … , *p*, we can then write

$$f(X|Z,\varphi )=f(X_{j}|X_{-j},Z,\varphi_{j})f(X_{-j}|Z,\varphi_{j},\varphi_{-j})$$

where $\varphi_{-j}=\varphi_{-j}(\varphi )$ is some function of φ. Suppose that for each *j* = 1, … , *p*,

$$f(X_{-j}|Z,\varphi_{j},\varphi_{-j})=f(X_{-j}|Z,\varphi_{-j})$$

Suppose further that there exists a prior f(φ) such that for each *j* = 1, … , *p*, the induced priors on $\varphi_{j}$ and $\varphi_{-j}$ factorise as

$$f(\varphi )=f(\varphi_{j},\varphi_{-j})=f(\varphi_{j})f(\varphi_{-j})$$

In the Gibbs sampler,^21^ we sample from each unknown component’s conditional distribution, given the observed data values, current values of other parameters and current imputations of missing values. Using the re-parametrisation of φ to $\left(\varphi_{j},\varphi_{-j}\right)$, we can derive the conditional distribution for $x_{j}^{\mathrm{mis}}$ used by the Gibbs sampler as

$$f(x_{j}^{\mathrm{mis}}|\psi ,\varphi_{j},\varphi_{-j},y,z,x_{j}^{\mathrm{obs}},x_{-j}^{\mathrm{mis}},x_{-j}^{\mathrm{obs}})\propto f(\psi ,\varphi_{j},\varphi_{-j})f(y,z,x_{j}^{\mathrm{mis}},x_{j}^{\mathrm{obs}},x_{-j}^{\mathrm{mis}},x_{-j}^{\mathrm{obs}}|\psi ,\varphi_{j},\varphi_{-j})\propto f(\psi )f(\varphi_{j})f(\varphi_{-j})f(y|x_{j}^{\mathrm{mis}},x_{j}^{\mathrm{obs}},x_{-j}^{\mathrm{mis}},x_{-j}^{\mathrm{obs}},z,\psi )f(x_{j}^{\mathrm{mis}},x_{j}^{\mathrm{obs}}|x_{-j}^{\mathrm{mis}},x_{-j}^{\mathrm{obs}},z,\varphi_{j})f(x_{-j}^{\mathrm{mis}},x_{-j}^{\mathrm{obs}}|z,\varphi_{-j})\propto f(y|x_{j}^{\mathrm{mis}},x_{j}^{\mathrm{obs}},x_{-j}^{\mathrm{mis}},x_{-j}^{\mathrm{obs}},z,\psi )f(x_{j}^{\mathrm{mis}},x_{j}^{\mathrm{obs}}|x_{-j}^{\mathrm{mis}},x_{-j}^{\mathrm{obs}},z,\varphi_{j})$$

where $x_{-j}^{\mathrm{obs}}$ denotes the observed values of $X_{-j}$ across all *n* subjects. This factorises into *n* independent densities of the form given in equation (4), as used by SMC-FCS. The conditional distribution for ψ used by the Gibbs sampler is given by

$$f(\psi |\varphi_{j},\varphi_{-j},y,z,x_{j}^{\mathrm{mis}},x_{j}^{\mathrm{obs}},x_{-j}^{\mathrm{mis}},x_{-j}^{\mathrm{obs}})\propto f(\psi )f(y|x_{j}^{\mathrm{mis}},x_{j}^{\mathrm{obs}},x_{-j}^{\mathrm{mis}},x_{-j}^{\mathrm{obs}},z,\psi )$$

which is the distribution used by SMC-FCS, as given in equation (5). The conditional distribution for $\varphi_{j}$ used by the Gibbs sampler is given by

$$f(\varphi_{j}|\psi ,\varphi_{-j},y,z,x_{j}^{\mathrm{mis}},x_{j}^{\mathrm{obs}},x_{-j}^{\mathrm{mis}},x_{-j}^{\mathrm{obs}})\propto f(\varphi_{j})f(x_{j}^{\mathrm{mis}},x_{j}^{\mathrm{obs}}|x_{-j}^{\mathrm{mis}},x_{-j}^{\mathrm{obs}},z,\varphi_{j})$$

which is the distribution used by SMC-FCS, as given in equation (6). The Gibbs sampler would then sample $\varphi_{-j}$ from its conditional distribution. However, since $\varphi_{-j}$ is not required for imputing missing values in *X_j_*, in SMC-FCS we do not need to sample values of $\varphi_{-j}$.

It follows that, under the stated conditions, at convergence the SMC-FCS algorithm gives draws of $x_{1}^{\mathrm{mis}},...,x_{p}^{\mathrm{mis}}$ from the Bayesian joint model defined by the models f(Y|X,Z,ψ) and f(X|Z,φ) and the priors f(ψ) and f(φ).
